# ﻿The bee genus *Anthidiellum* in Vietnam: descriptions of five new species and the first male of *Anthidiellumcoronum* (Hymenoptera, Megachilidae)

**DOI:** 10.3897/zookeys.1144.98644

**Published:** 2023-02-02

**Authors:** Ngat Thi Tran, Michael S. Engel, Cuong Quang Nguyen, Duong Dinh Tran, Lien Thi Phuong Nguyen

**Affiliations:** 1 Institute of Ecology and Biological Resources, Vietnam Academy of Science and Technology, 18 Hoang Quoc Viet Road, Nghia Do, Cau Giay, Hanoi, Vietnam; 2 Graduate University of Science and Technology, Vietnam Academy of Science and Technology, 18 Hoang Quoc Viet Road, Nghia Do, Cau Giay, Hanoi, Vietnam; 3 Division of Invertebrate Zoology, American Museum of Natural History, Central Park West at 79; 4 th; 5 Street, New York, New York 10024-5192, USA; 6 Division of Entomology, Natural History Museum, and Department of Ecology and Evolutionary Biology, 1501 Crestline Drive – Suite 140, University of Kansas, Lawrence, Kansas 66045-4415, USA

**Keywords:** Apoidea, Anthidiini, Megachilinae, morphology, taxonomy

## Abstract

The Vietnamese fauna of bees in the *Anthidiellum* Cockerell (Megachilinae, Anthidiini) is reviewed. Seven species are recognized, representing two subgenera. Five new species are described and figured as: Anthidiellum (Clypanthidium) nahang Tran, Engel & Nguyen, **sp. nov.**, A. (Pycnanthidium) ayun Tran, Engel & Nguyen, **sp. nov.**, A. (P.) chumomray Tran, Engel & Nguyen, **sp. nov.**, A. (P.) flavaxilla Tran, Engel & Nguyen, **sp. nov.**, and A. (P.) cornu Tran, Engel & Nguyen, **sp. nov.** from the northern and central highlands of Vietnam. Two previously described species are newly recorded for the fauna: A. (P.) carinatum (Wu) and A. (P.) coronum (Wu), with the male of the latter species described and illustrated for the first time. An identification key is provided for all species of *Anthidiellum* occurring in Vietnam.

## ﻿Introduction

The genus *Anthidiellum* Cockerell (Anthidiini) consists of typically small, robust, megachiliform to somewhat bombiform bees. It can be separated from other genera by the following characteristics: body small, robust, metasoma oval-rounded, with strongly developed omaular carinae/lamellae, and an often-lamellate pronotal lobe (Michener 2007). Species are frequently black, often with maculae laterally or with unbroken bands on the terga (see species of *Ranthidiellum* Pasteels: Engel 2009; Nalinrachatakan et al. 2021). Additionally, the juxtantennal carinae are absent; the subantennal sulci are outwardly arcuate; the axillae are rounded, without a spine; the mesoscuto-mesoscutellar sulcus is broad; the mesoscutellum lacks lateral spines, but typically extends posteriorly as a broad, thin, truncate, or medially emarginate lamella overhanging the metanotum and propodeum; behind the propodeal spiracle there is a fovea, delimited by a carina, although sometimes the fovea is no larger than the spiracle; the jugal lobe of the hind wing is less than one-half as long as the vannal lobe; cu-v in the hind wing is less than one-half as long as 2M+Cu; the arolia are well developed; the mandible of the female has three or four teeth (tri- or quadridentate), while the male has two or three teeth (bi- or tridentate) (Michener 2007).

Currently, the genus *Anthidiellum* comprises nearly 65 described species in seven subgenera, most of which are found in the Old World (Pasteels 1972; Michener and Griswold 1994; Griswold 2001; Wu 2004; Michener 2007; Engel 2009; Niu et al. 2016; Soh et al. 2016; Kumar et al. 2017; Nalinrachatakan et al. 2021). There are about 30 species known from Asia, and about half of the species occur in Southeast Asia, all of which belong to the subgenera *Clypanthidium* Pasteels, *Pycnanthidium* Krombein, and *Ranthidiellum* Pasteels.

In Vietnam, only two prior records of the genus were known from northern provinces (Khuat et al. 2012). However, upon re-examination, the specimens of Khuat et al. (2012) are misidentified, one belonging to *Heriades* Spinola (Osmiini) and the material of the other now missing. Recently, five new species and two newly recorded species of *Anthidiellum* were discovered in the northern and central highlands of Vietnam. Herein, we describe the five new species, as well as the first male of Anthidiellum (Pycnanthidium) coronum (Wu, 2004). Illustrations and a key are also given to all species of the genus from Vietnam. Table 1 summarizes the species of *Anthidiellum* currently known from Vietnam, with information on the known sexes and distribution.

**Table 1.** Summary of species currently in the genus *Anthidiellum* Cockerell from Vietnam.

Species	Sexes known	Distribution
Anthidiellum (Clypanthidium) nahang Tran, Engel & Nguyen, sp. nov.	♀	Tuyen Quang
Anthidiellum (Pycnanthidium) ayun Tran, Engel & Nguyen, sp. nov.	♀	Gia Lai
Anthidiellum (Pycnanthidium) carinatum (Wu, 1962) *	♀♂	Son La, Vinh Phuc, Kon Tum, Gia Lai, Dak Lak
Anthidiellum (Pycnanthidium) chumomray Tran, Engel & Nguyen, sp. nov.	♀	Kon Tum
Anthidiellum (Pycnanthidium) flavaxilla Tran, Engel & Nguyen, sp. nov.	♀	Gia Lai
Anthidiellum (Pycnanthidium) cornu Tran, Engel & Nguyen, sp. nov.	♂	Kon Tum
Anthidiellum (Pycnanthidium) coronum (Wu, 2004)**	♀♂	Gia Lai

* Newly recorded species. ** Newly recorded species and the first male described.

## ﻿Materials and methods

Specimens examined in the present study are deposited in the collection of Hymenoptera of the Institute of Ecology and Biological Resources (**IEBR**), Hanoi, Vietnam and Division of Invertebrate Zoology, American Museum of Natural History, New York, USA (**AMNH**). Adult morphological and color characters were examined with a Nikon SMZ745 stereomicroscope, while images were photographed with a Nikon SMZ800N digital stereomicroscope, and with an attached ILCE-5000L/WAP2 digital camera. Stacked focus images were prepared using Helicon Focus 7. Lastly, all files were processed with Adobe Photoshop CS6. The morphological terminology used in the descriptions follows Engel (2001) and Michener (2007), with certain body metrics following those of Niu et al. (2004): **body length**: measured from the base of the antennal torulus to metasomal apex (in dorsal view), **head length**: measured from the medioapical margin of the clypeus to the upper margin of the vertex (in facial view), **head width**: measured at the widest point of the head across the compound eyes (in facial view), **eye width**: the greatest width of the compound eye (in profile), **genal width**: the greatest width of the gena (in profile), **intertegular distance**: measured between the inner rims of the tegulae (in dorsal view). The abbreviations F, S, and T (followed by Arabic numerals) refer to numbered flagellomeres, metasomal sterna, and metasomal terga, respectively. Additional abbreviations: **NP**, National Park; **RS**, Ranger Station.

## ﻿Systematics

### ﻿Genus *Anthidiellum* Cockerell, 1904

**Subgenus Clypanthidium Pasteels, 1968**

#### ﻿ Anthidiellum (Clypanthidium) nahang

Taxon classificationAnimaliaHymenopteraMegachilidae

Tran, Engel & Nguyen sp. nov.

6E0932A3-5DED-5780-8A62-553A660CD648

https://zoobank.org/FC9CA845-4F28-42B4-B4B4-0171FB79020D

Fig. 1A–F

##### Type material.

***Holotype*.** Vietnam: ♀, Tuyen Quang, Na Hang, v.2018 [May 2018], malaise trap, Long Dang Khuat leg. [IEBR].

##### Diagnosis.

The female of this species is most similar to that of A. (Clypanthidium) popovii (Wu, 1962), as both have the mandible much broader apically than basally; metatibia and metabasitarsus both without a longitudinal carina on the prolateral surface; metabasitarsus slender, width much less than greatest width of metatibia; forewing with veins bicolorous; and metasomal T1–T3 black. The new species can be distinguished from the female of the latter species by the following: mandible with interspace between the first two teeth broad, about 2× the interspace between the second and third tooth (mandible with interspace of the first two teeth narrow, subequal to that between the second and third tooth in *A.popovii*), mesoscutum with postero-lateral corner forming an obtuse angle (mesoscutum with postero-lateral corner forming a nearly right angle in *A.popovii*), clypeus with small yellow markings laterally (clypeus without yellow markings laterally in *A.popovii*), forewing rather transparent basally and dark brown distally (forewing dark basally and yellow distally in *A.popovii*), metasomal T4 black and T5–T6 yellow (metasomal T4–T5 yellow, T6 black in *A.popovii*).

##### Description.

♀: body length (holotype) 9.5 mm, forewing length (holotype) 9 mm.

**Figure 1.**
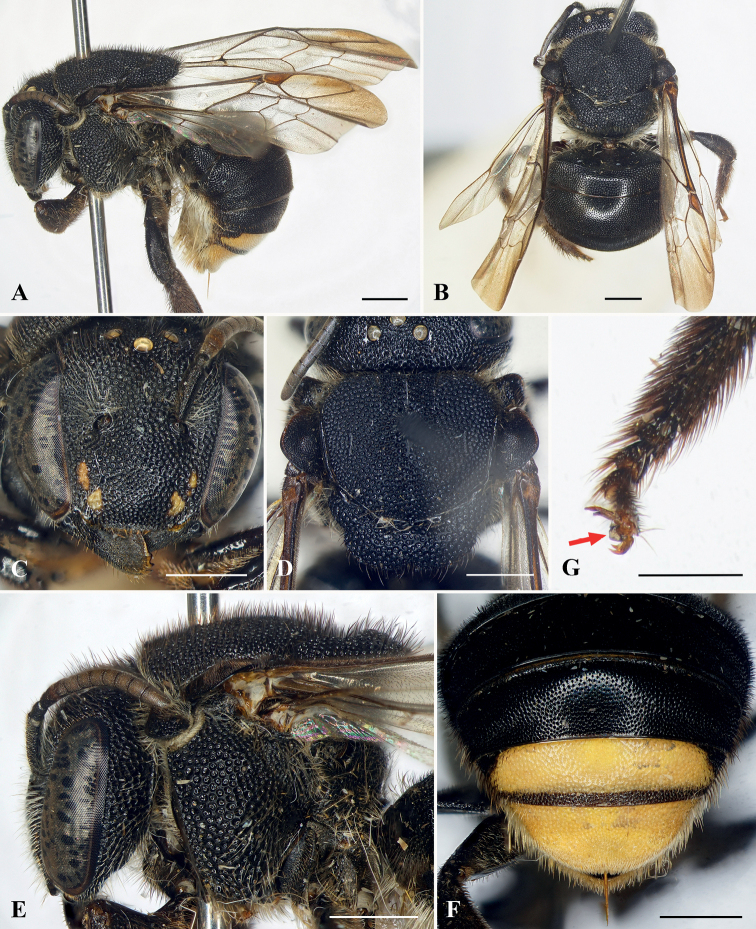
Anthidiellum (Clypanthidium) nahang Tran, Engel & Nguyen, sp. nov., holotype, female **A** lateral habitus **B** dorsal habitus **C** facial view **D** mesosoma in dorsal view **E** head and mesosoma in profile **F** apical metasomal terga **G** metapretarsus, red arrow indicating arolium. Scale bars: 1 mm (**A–F**); 0.5 mm (**G**).

***Structure*.** Head broader than long, approximately 1.2× as broad as long (Fig. 1C). Compound eyes 2.8× as long as broad, 1.5× genal width. Mandible quadridentate, interspace between first two teeth broad, about 2× that of interspace between second and third tooth (Fig. 1C). Clypeus slightly convex medially, about 1.9× as broad as long. Supraclypeal area slightly convex medially. Scape slender, about 4.5× as long as broad; pedicel approximately as broad as long and 1.1× length of F1; F1 as broad as long and subequal to length of F2+F3; F4–F6 longer and subequal in length, longer than F3; F7–F9 subequal in length, longer than F4; F10 longest flagellomere, about 1.6× as long as broad. Mesosoma approximately 1.2× as broad as long; mesoscutum with postero-lateral corner forming an obtuse angle; mesoscutellum short, convex in dorsal view and emarginate medio-apically (Fig. 1D). Forewing with two submarginal cells, first submarginal cell broader than second submarginal cell. Metapretarsus with arolium (Fig. 1G). Metasoma curved ventrally, giving metasoma arched appearance in profile (Fig. 1A).

***Sculpturing and texture*.** Mandible with small, dense, nearly contiguous punctures. Clypeus with round, dense, nearly contiguous punctures of unequal sizes, puncture sizes larger laterally than those on disc (Fig. 1C). Supraclypeal area and paraocular area with round, dense, nearly contiguous punctures, puncture sizes larger and rougher than on clypeus. Vertex with large, coarse, dense, contiguous or nearly contiguous punctures. Mesoscutum with round, distinct, dense, nearly contiguous punctures; mesepisternum with punctures as on mesoscutum; mesoscutellum with punctures as on mesoscutum except some slightly larger and coarser (Fig. 1D, E). Metasomal T1–T4 with round, small, dense punctures, T5–T6 with wrinkled punctures.

***Color*.** Body black except as follows: clypeus with yellow markings laterally, paraocular area with yellow markings baso-laterally; mandible with yellowish-brown apex (Fig. 1C); forewing rather transparent basally and dark brown apically (Fig. 1A, B); metasomal T5 yellow except black baso-laterally and on apical margin, T6 yellow (Fig. 1F).

***Pubescence*.** Paraocular area with short, sparse, white setae. Clypeus with short, sparse, white setae laterally and some short yellowish setae apically. Antennal scape with short, yellowish setae; face above antennal torulus with diffuse tufts of white setae (Fig. 1C). Frons with short white setae. Vertex with short blackish setae. Mesoscutum with short, dense, blackish setae on dorsal surface and yellowish setae laterally, mesoscutellum with short, dense, blackish setae. Meso- and metatrochanters with dense, yellowish setal tufts ventrally; prolateral surface of mesotibia with short brownish setae, retrolateral surface of mesotibia and both pro- and retrolateral surfaces of mesobasitarsus with long brownish setae; prolateral surface of metatibia with short blackish setae; retrolateral surface of metatibia with short brownish setae; both pro- and retrolateral surfaces of metabasitarsus with long blackish setae. Metasomal T1–T4 with short blackish setae; T5–T6 with short yellowish setae; T6 apically with short, soft, feathery, yellowish white setae (Fig. 1F); S2–S6 with long, dense, white to yellowish scopal setae (Fig. 1A).

♂: Latet.

##### Etymology.

The specific epithet is a toponym for the type locality, the Na Hang district in Tuyen Quang Province. The name is treated as a noun in apposition.

###### ﻿Subgenus Pycnanthidium Krombein, 1951

Based on the presence or absence of a longitudinal carina on the prolateral surface of the metatibia and metabasitarsus and the normal or enlarged metabasitarsus of the female, *Pycnanthidium* can be divided into two distinctive groups: the first group with nine species includes *A.carinatum* (Wu), *A.biroi* (Friese), *A.forstenii* (Ritsema), *A.nigriceps* (Friese), *A.riparium* (Cockerell), *A.smithii* (Ritsema), *A.ayun* Tran, Engel & Nguyen, sp. nov., *A.chumomray* Tran, Engel & Nguyen, sp. nov., and *A.flavaxilla* Tran, Engel & Nguyen, sp. nov., while the second group, with a similar number of species, includes *A.butarsis* Griswold, *A.coronum* (Wu), *A.krombeini* Griswold, *A.latipes* (Bingham), *A.melanaspis* Cockerell, *A.ramakrishnae* (Cockerell), *A.rasorium* (Smith), *A.turneri* (Friese), and *A.cornu* Tran, Engel & Nguyen, sp. nov.

#### ﻿ Anthidiellum (Pycnanthidium) ayun

Taxon classificationAnimaliaHymenopteraMegachilidae

Tran, Engel & Nguyen sp. nov.

8B8108AE-254D-57BB-8C11-5FE6F05F0C94

https://zoobank.org/4CF71E2F-C15B-457A-B383-5C3BF9AC34B1

Fig. 2A–H

##### Type material.

***Holotype*.** Vietnam: ♀, Gia Lai, Mang Yang, Ayun, Kon Ka Kinh NP, 14°13'18"N, 108°19'02"E, alt. 907 m, 26.iii.2022 [26 March 2022], Lien Thi Phuong Nguyen, Ngat Thi Tran, Cuong Quang Nguyen leg. [IEBR].

***Paratypes*.** Vietnam: 51♀♀, same data as holotype [41♀♀ in IEBR; 10♀♀ in AMNH]; 30♀♀, Gia Lai, Mang Yang, Ayun, Kon Ka Kinh NP, 14°12'11"N, 108°18'58"E, alt. 834 m, 25.iii.2022 [25 March 2022], Lien Thi Phuong Nguyen, Ngat Thi Tran, Cuong Quang Nguyen leg.; 4♀♀, Gia Lai, Mang Yang, Ayun, Kon Ka Kinh NP, 14°12'11"N, 108°18'58"E, alt. 834 m, 26.iv.2022 [25 April 2022], Lien Thi Phuong Nguyen, Ngat Thi Tran leg. [IEBR].

##### Diagnosis.

This female of this species is most similar to A. (P.) chumomray (*vide infra*), sharing with it the following characters: metatibia and metabasitarsus with a longitudinal carina on prolateral surfaces, and metasomal T2, supraclypeal area, pronotal lobe, and axillae black. It can be separated from that species easily by the following characters: mandible tridentate, teeth gradually longer and teeth sharp apically; clypeus approximately 1.4× as broad as long and with trapezoidal yellow marking medially; mesoscutellum convex and bigibbous; paraocular area black.

##### Description.

♀: body length 7.0–7.5 mm (holotype = 7.5 mm). Forewing length 6.5–7.0 mm (holotype = 7.0 mm).

**Figure 2.**
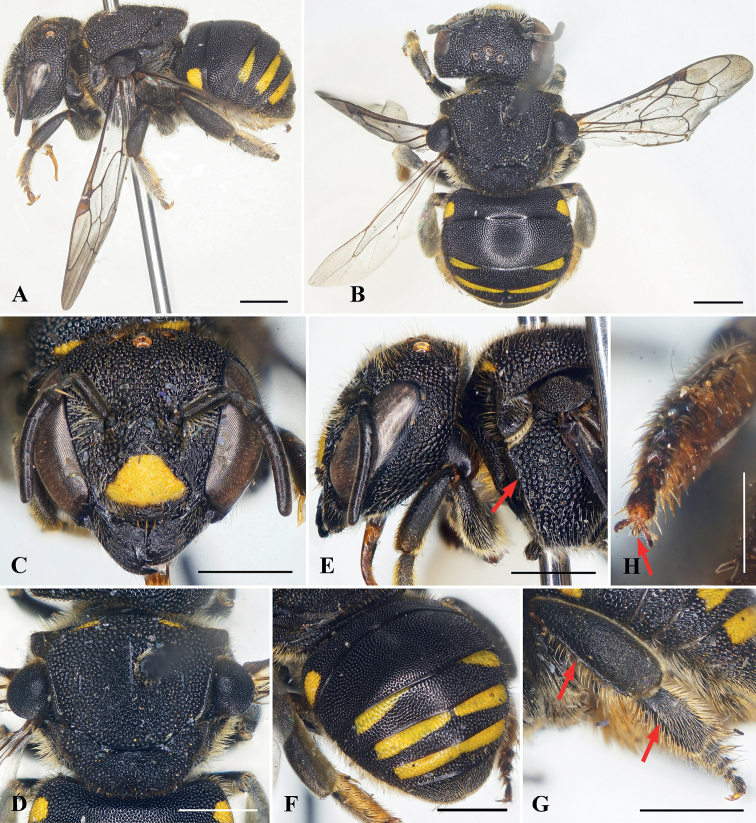
Anthidiellum (Pycnanthidium) ayun Tran, Engel & Nguyen, sp. nov., holotype, female **A** dorsolateral oblique habitus **B** dorsal habitus **C** facial view **D** mesosoma in dorsal view **E** head and anterior mesosoma in profile, red arrow indicating omaular carina **F** dorsal oblique view of metasoma **G** prolateral surfaces of metatibia and metatarsus, with red arrows indicating longitudinal carinae **H** metapretarsal arolium (red arrow). Scale bars: 1 mm (**A–G**); 0.5 mm (**H**).

***Structure*.** Head broader than long, approximately 1.2× as broad as long (Fig. 2C). Compound eyes about 2.3× as long as broad, about 1.4× genal width. Mandible tridentate, teeth gradually longer and sharp apically, interspace between first two teeth broad, second tooth nearly contiguous with third tooth (Fig. 2C). Clypeus slightly convex medially, approximately 1.4× as broad as long. Supraclypeal area slightly convex medially. Scape slender, about 3.1× as long as broad, pedicel approximately 1.5× as long as broad and about 1.3× length of F1; F1 as long as broad and 1.5× length of F2; F3–F5, F6–F7, F8–F9 subequal in length; F10 longest flagellomere, about 1.5× as long as broad. Mesosoma approximately 1.2× as broad as long; mesoscutum with medioapical margin slightly sharp; mesoscutellum convex, with mediolongitudinal depression apically giving bigibbous surface (Fig. 2D). Prolateral surface of metatibia and metabasitarsus each with a longitudinal carina (Fig. 2G). Metapretarsal arolium present (Fig. 2H).

***Sculpturing and texture*.** Outer surface of mandible with abundant, small, dense punctures except larger, wrinkled punctures on proximal half. Clypeus with dense, shallow punctures, puncture much smaller on apical margin than on disc. Paraocular area, supraclypeal area, and frons with large, coarse, contiguous punctures. Vertex with round, dense punctures of unequal sizes, puncture sizes smaller than those on frons. Mesoscutum with large, coarse, contiguous punctures; mesoscutellum with contiguous punctures of unequal sizes, puncture sizes smaller than those of mesoscutum (Fig. 2D). Metasomal T1–T6 with small, round, dense punctures except punctures larger laterally.

***Color*.** Body black except as follows: clypeus with trapezoid yellow marking except lateral and apical margins black (Fig. 2C). Mesoscutum with short, thin yellow markings latero-anteriorly (Fig. 2D). Metasomal T1 with yellow markings laterally; T3 basally with yellow band and large interrupted medially; T4 basally with yellow band and narrow interrupted medially (near as continuously in some paratypes); T5 basally with yellow band (Fig. 2A, B, F).

***Pubescence*.** Ventral margin of mandible with abundant, short, yellowish setae intermixed with long setae. Clypeus with short yellowish setae except sparse, erect, yellow setae on apical margin. Paraocular area, supraclypeal area, frons, and vertex with short yellowish setae. Diffuse tufts of long, dense, plumose, yellowish setae above antennal toruli. Mesoscutum and mesoscutellum with abundant short yellowish setae; mesoscutellum with long, erect, yellowish setae ventro-apically; propodeum with long, plumose, dense, yellowish setae. Prolateral surfaces of basitarsi and tarsi with dense, erect, yellowish setae; retrolateral surfaces of these same podites with tawny yellow setae. Metasomal T1–T6 with short, sparse, yellowish setae except short, yellowish setae on T6 apically; S2–S5 with long, dense, tawny yellow scopal setae.

♂: Latet.

##### Etymology.

The specific epithet is a toponym for type locality, the Ayun commune in Gia Lai Province. The name is treated as a noun in apposition.

#### ﻿ Anthidiellum (Pycnanthidium) carinatum

Taxon classificationAnimaliaHymenopteraMegachilidae

(Wu, 1962)

38B14821-E0C4-5BEB-B46C-83C26C619DA0

Figs 3A–G , 4A–F

Paraanthidium carinatum Wu, 1962: 165 [holotype ♂, paratype ♀]. Wu et al. 1988: 61.
Trachusa (Paraanthidium) carinatum (Wu): Wu 2006: 181.
Anthidiellum (Pycnanthidium) carinatum (Wu): Niu et al. 2016: 337.

##### Material examined.

Vietnam: 2♀♀, Son La, Moc Chau, Nam Kham, alt. 630 m, 22.vi.2020 [22 June 2020], Lien Thi Phuong Nguyen, Cuong Quang Nguyen, Ngat Thi Tran, Thai Van Mai leg. [1♀ in IEBR, 1♀ in AMNH] ; 1♀; Son La, Moc Chau, 27.vi.2022, Lien Thi Phuong Nguyen, Lam Xuan Truong, Cuong Quang Nguyen, Ngat Thi Tran leg.,; 1♀, Vinh Phuc, Me Linh station, 12.vi.2018 [12 June 2018], Phong Huy Pham leg.; 3♂♂, Kon Tum, Sa Thay, Chu Mom Ray NP, 14°47'24.5"N, 107°59'46.5"E, alt. 729 m, 25.iv.2016 [25 April 2016], Lien Thi Phuong Nguyen, Dai Dac Nguyen, Ngat Thi Tran leg. [IEBR]; 6♂♂, Kon Tum, Sa Thay, Chu Mom Ray NP, Ro Koi RS, 14°27'25"N, 107°36'22"E, alt. 267 m, 25.iv.2022 [25 June 2022], Lien Thi Phuong Nguyen, Ngat Thi Tran leg. [4♂♂ in IEBR, 2♂♂ in AMNH]; 3♀♀, Gia Lai, Mang Yang, Ayun, Kon Ka Kinh NP, 14°13'18"N, 108°19'02"E, alt. 907 m, 26.iii.2022 [26 March 2022], Lien Thi Phuong Nguyen, Ngat Thi Tran, Cuong Quang Nguyen leg.; 1♀, Gia Lai, Mang Yang, Ayun, Kon Ka Kinh NP, 14°12'11"N, 108°18'58"E, alt. 834 m, 25.iii.2022 [25 March 2022], Lien Thi Phuong Nguyen, Ngat Thi Tran, Cuong Quang Nguyen leg.; 1♂, Dak Lak, Krong Bong, Krong Kmar, Chu Yang Sin NP, 12°28'46.4"N, 108°13'46.4"E, alt. 739 m, 2.v.2016 [2 May 2016], Lien Thi Phuong Nguyen, Dai Dac Nguyen, Ngat Thi Tran leg. [IEBR].

##### Remarks.

This species can be separated from the similar species *A.smithii* (Ritsema) by the following combination of traits: punctures of mesoscutum mesad parapsidal line dense but not contiguous; axilla laterally not reaching tangent of lateral margin of mesoscutum; and clypeus more densely, finely punctate, and posterior margin of gena not as strongly carinate posteriorly.

##### Nesting biology.

**Figure 3.**
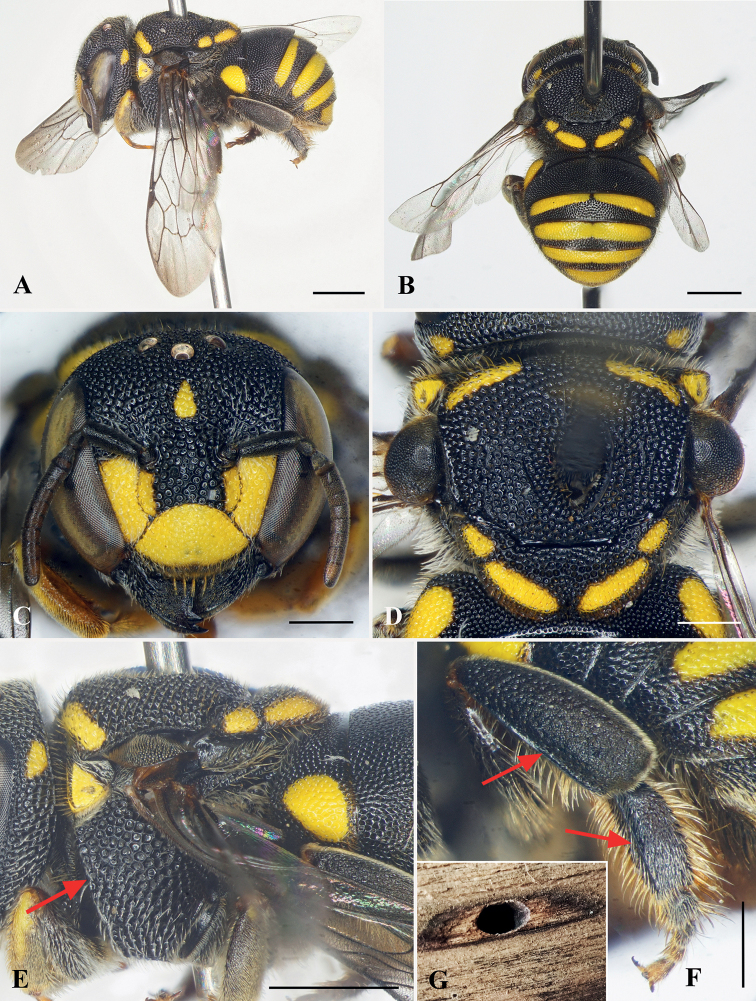
Anthidiellum (Pycnanthidium) carinatum (Wu, 1962), female **A** lateral habitus **B** dorsal-posterior habitus **C** facial view **D** mesosoma in dorsal view **E** mesosoma in lateral view, red arrow indicating omaular carina **F** prolateral surfaces of metatibia and metatarsus, with red arrows indicating longitudinal carinae **G** nest entrance. Scale bars: 1 mm (**A, B, E**); 0.5 mm (**C, D, F**).

A nest was found in an unused wooden plank in a warehouse in Son La Province. The entrance hole of the nest was oval-shaped, with a length and a width of about 4 mm and 3 mm, respectively (Fig. 3G). A female was observed and collected when flying out from the nest.

**Figure 4.**
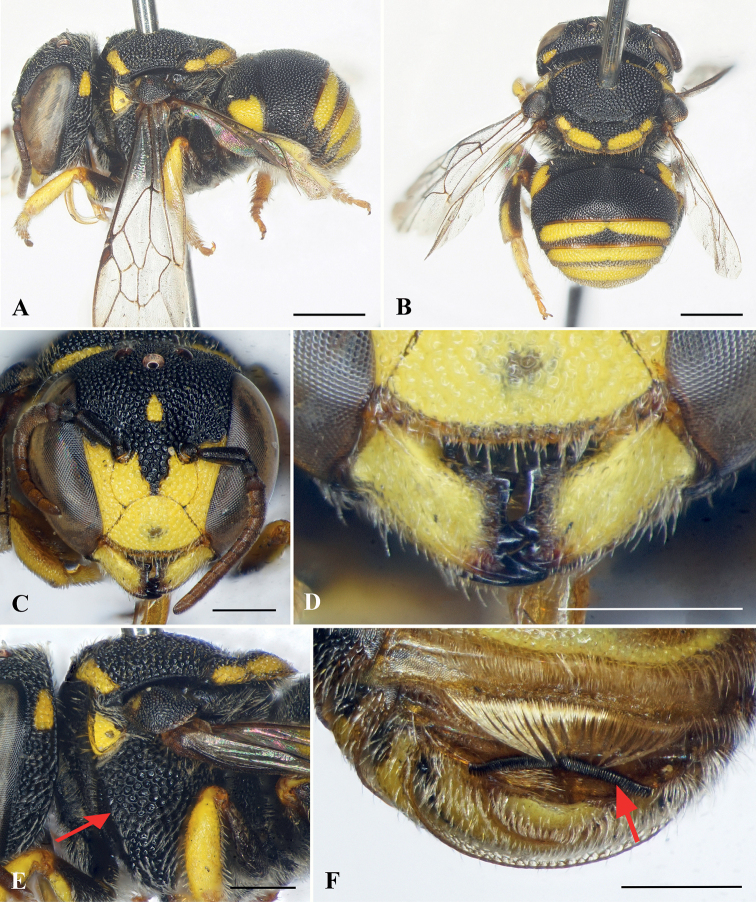
Anthidiellum (Pycnanthidium) carinatum (Wu, 1962), male **A** lateral habitus **B** dorsal-posterior habitus **C** facial view **D** mandibles **E** mesosoma in lateral view, red arrow indicating omaular carina **F** apical metasomal sterna, red arrow indicating apical comb. Scale bars: 1 mm (**A, B**); 0.5 mm (**C–F**).

#### ﻿ Anthidiellum (Pycnanthidium) chumomray

Taxon classificationAnimaliaHymenopteraMegachilidae

Tran, Engel & Nguyen sp. nov.

AD37E79B-2C76-5FE8-9D9A-E2F1B7C146F2

https://zoobank.org/B5C89018-9425-4E1B-8FE7-99D66A56807D

Fig. 5A–G

##### Type material.

***Holotype*.** Vietnam: ♀, Kon Tum, Sa Thay, Sa Son, Chu Mom Ray NP, 14°25'19"N, 107°43'54"E, alt. 653 m, 24.iv.2022 [24 April 2022], Lien Thi Phuong Nguyen, Ngat Thi Tran leg. [IEBR].

##### Diagnosis.

The female of this species is most similar to *A.ayun* (*vide supra*). It can be separated from that species by the tridentate mandible and in which the teeth are subequal in length not sharply pointed; by the clypeus approximately 1.6× as broad as long and with a subhexagonal yellow mark medially; the flat mesoscutellum; and the paraocular area with yellow extending along the inner ocular margin to the lower antennal torular tangent.

##### Description.

♀: body length (holotype) 6.5 mm. Forewing length (holotype) 6.0 mm.

**Figure 5.**
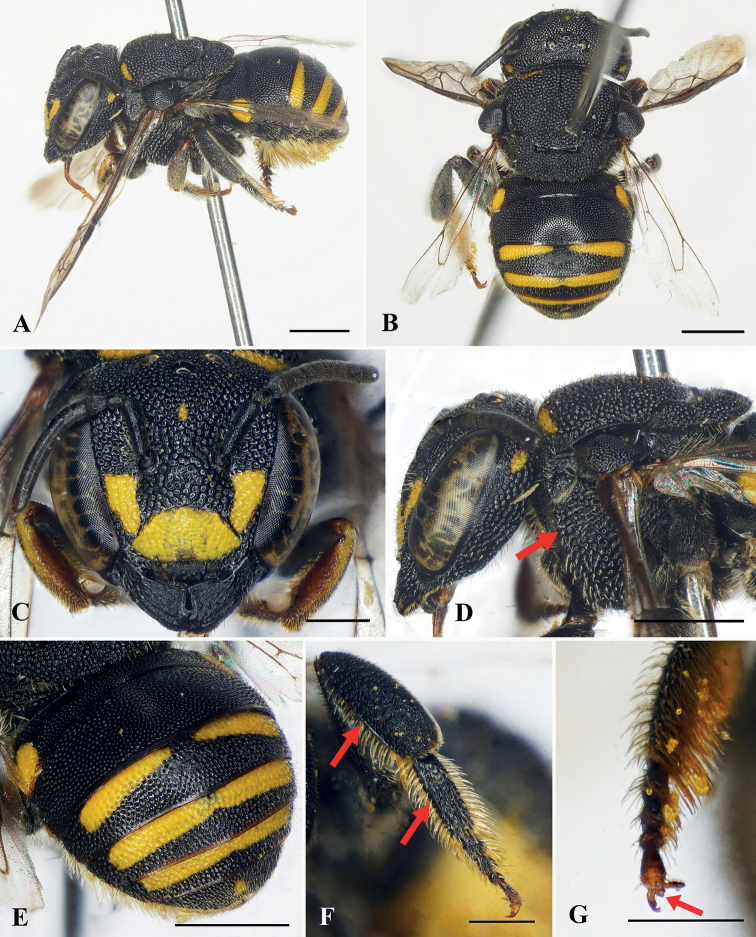
Anthidiellum (Pycnanthidium) chumomray Tran, Engel & Nguyen, sp. nov., holotype, female. **A** slightly dorsal oblique lateral habitus **B** dorsal habitus **C** facial view **D** head and mesosoma in lateral view, red arrow indicating omaular carina **E** dorsal oblique view of metasoma **F** prolateral surfaces of metatibia and metatarsus, with red arrows indicating longitudinal carinae **G** metatarsus and metapretarsus, red arrow indicating arolium. Scale bars: 1 mm (**A, B, D, E**); 0.5 mm (**C, F, G**).

***Structure*.** Head broader than long, approximately 1.2× as broad as long (Fig. 5C). Compound eyes about 2.4× as long as broad, 1.6× genal width. Mandible tridentate, teeth subequal in length, interspace between first two teeth broad, second tooth nearly contiguous with third tooth (Fig. 5C). Clypeus slightly convex medially, about 1.6× as broad as long. Supraclypeal area slightly convex medially. Scape slender, about 3.2× as long as broad, pedicel about 1.4× as long as broad and about 1.6× length of F1, F1 as long as broad, approximately 1.3× length of F2; F2–F3 subequal in length, F4–F9 subequal in length, longer than F2, F10 longest flagellomere, about 1.6× as long as broad. Mesosoma approximately 1.1× as broad as long; mesoscutal medio-apical margin vertical; mesoscutellum relatively flat, with mediolongitudinal depression apically (Fig. 5B). Forewing with two submarginal cells, first submarginal cell broader than second submarginal cell. Prolateral surface of metatibia and metabasitarsus with longitudinal carinae (Fig. 5F). Metapretarsal arolium present (Fig. 5G).

***Sculpturing and texture*.** Outer surface of mandible with small, wrinkled punctures. Clypeus with shallow, dense punctures, apical margin with puncture sizes much smaller than disc. Paraocular area and supraclypeal area with large, coarse, contiguous punctures (Fig. 5C). Frons and vertex with dense punctures of unequal sizes, puncture sizes smaller than those on clypeus. Mesoscutum with large, contiguous punctures, except polished and impunctate on vertical medio-apical margin; mesoscutellum with contiguous punctures, puncture sizes smaller than those on mesoscutum (Fig. 5B). Metasomal T1–T6 with small, round, dense punctures except punctures larger laterally.

***Color*.** Body black except as follows: paraocular area with yellow extending from base to lower antennal torular tangent. Clypeus with subhexagonal yellow mark medially, apical margin black. Frons with small yellow spot (Fig. 5C). Gena with short yellow markings near upper posterior margin of compound eye (Fig. 5A). Mesoscutum with short, thin, transverse, yellow markings latero-anteriorly along anterior border (Fig. 5A, B). Profemur apically and prolateral surface of antero-vertical half of protibia with brownish yellow marking; mesotibia with small yellow marking basally and brown on prolateral surface of antero-vertical half. Metasomal T1 with yellow laterally; T3 with basal yellow band, with narrow interruption medially; T4–T5 basally with yellow bands; T6 with small yellow mark medially (Fig. 5A, B, E).

***Pubescence*.** Ventral margin of mandible with abundant short, yellowish setae intermixed with some long setae. Clypeus, paraocular area, supraclypeal area, frons, and vertex with abundant short, sparse, yellowish setae. Tufts of long, plumose, yellowish setae above antennal toruli. Mesoscutum and mesoscutellum with abundant short yellowish setae; mesoscutellum with long, erect, yellow setae arising from beneath apical margin; propodeum with long, plumose, yellowish setae. Prolateral surfaces of basitarsi and tarsi with dense, erect, yellowish setae; retrolateral surfaces of these same podites with tawny yellow setae. Metasomal T1–T2 nearly bare, T3–T6 with short, sparse, yellowish setae except dense, yellowish setae on T6 apically; S2–S5 with long, dense, yellowish scopal setae.

♂: Latet.

##### Etymology.

The specific epithet is a toponym for the type locality, Chu Mom Ray National Park in Kon Tum Province. The name is treated as a noun in apposition.

#### ﻿ Anthidiellum (Pycnanthidium) flavaxilla

Taxon classificationAnimaliaHymenopteraMegachilidae

Tran, Engel & Nguyen sp. nov.

B438F682-0FA6-503C-8D7C-F992F2766201

https://zoobank.org/30D3EF30-64BA-45CA-A1F2-442C118BA7DD

Fig. 6A–G

##### Type material.

***Holotype*.** Vietnam: ♀, Gia Lai, Mang Yang, Ayun, Kon Ka Kinh NP, 14°13'18"N, 108°19'02"E, alt. 907 m, 26.iii.2022 [26 March 2022], Lien Thi Phuong Nguyen, Ngat Thi Tran, Cuong Quang Nguyen leg. [IEBR].

***Paratypes*.** Vietnam: 3♀♀, same data as holotype [1♀ in IEBR; 2♀♀ in AMNH]; 2♀♀, Gia Lai, Mang Yang, Ayun, Kon Ka Kinh NP, 14°12'11"N, 108°18'58"E, alt. 834 m, 25.iii.2022 [25 March 2022], Lien Thi Phuong Nguyen, Ngat Thi Tran, Cuong Quang Nguyen leg.; 1♀, Gia Lai, Mang Yang, Ayun, Kon Ka Kinh NP, 14°12'11"N, 108°18'58"E, alt. 834 m, 26.iv.2022 [26 April 2022], Lien Thi Phuong Nguyen, Ngat Thi Tran leg.; 1♀, Gia Lai, Mang Yang, Ayun, Kon Ka Kinh NP, 14°12'11"N, 108°18'58"E, alt. 834 m, 27.iv.2022 [27 April 2022], Lien Thi Phuong Nguyen, Ngat Thi Tran leg.; 1♀, Gia Lai, Mang Yang, Ayun, Kon Ka Kinh NP, 14°12'11"N, 108°18'58"E, alt. 834 m, 28.iv.2022 [28 April 2022], Lien Thi Phuong Nguyen, Ngat Thi Tran leg. [IEBR].

##### Diagnosis.

The female of this species is most similar to that of A. (P.) carinatum (Wu 1962) as both have the metatibia and metabasitarsus each with a longitudinal carina on the prolateral surface; tridentate mandibles, with the teeth sharp apically; similar yellow markings on the face; and metasomal T2 black. The new species can be distinguished in the female from the latter species by the following: mesoscutum with short anterior-facing surface at medioapical margin, with anterior surface polished and impunctate (mesoscutum nearly straight medioapically, with dense punctures just before margin in *A.carinatum*); mesoscutellum black, strongly convex, with mediolongitudinal depression apically and giving apical surface a weakly bigibbous appearance (mesoscutellum nearly flat and yellow to yellowish brown on margin apicolaterally in *A.carinatum*).

##### Description.

♀: body length 6.5–7.0 mm (holotype = 7.0 mm), forewing length 6.0–6.2 mm (holotype = 6.2 mm).

**Figure 6.**
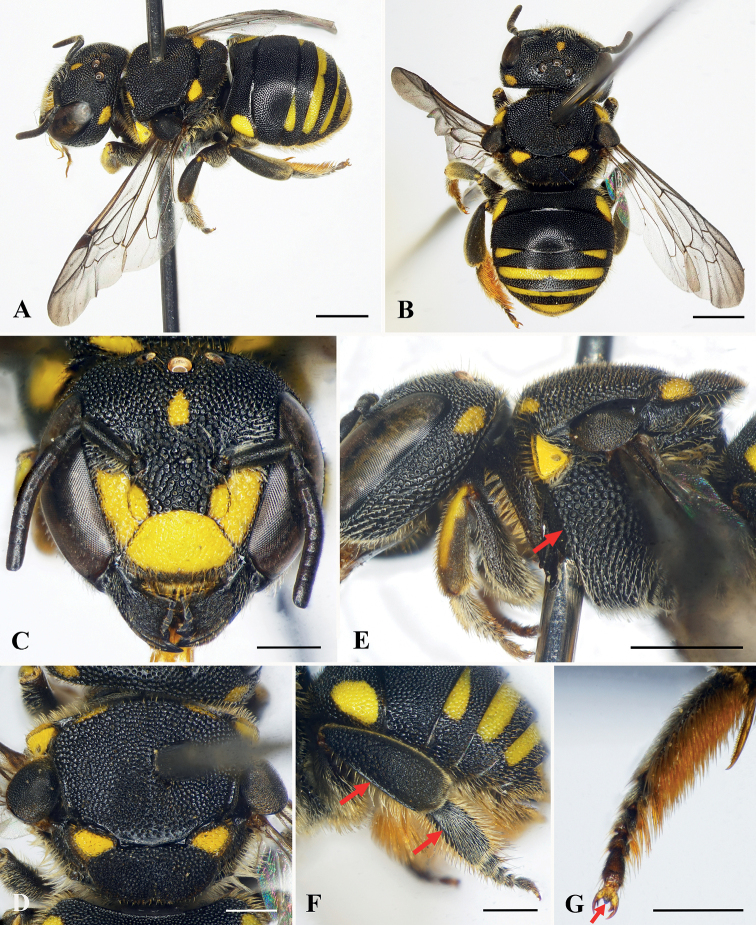
Anthidiellum (Pycnanthidium) flavaxilla Tran, Engel & Nguyen, sp. nov., holotype, female **A** dorsolateral oblique habitus **B** dorsal habitus **C** facial view **D** mesosoma in dorsal view **E** head and mesosoma in profile, red arrow indicating omaular carina **F** prolateral surfaces of metatibia and metatarsus, with red arrows indicating longitudinal carinae **G** metatarsus and metapretarsus, red arrow indicating metapretarsal arolium. Scale bars: 1 mm (**A, B, D, E**); 0.5 mm (**C, F, G**).

***Structure*.** Head broader than long, about 1.2× as broad as long (Fig. 6C). Compound eyes about 2.1× as long as broad and 1.4× genal width. Mandible tridentate, teeth gradually longer, interspace between first two teeth broad, second tooth contiguous with third tooth (Fig. 6C). Clypeus slightly convex medially, about 1.6× as broad as long. Supraclypeal area slightly convex medially. Scape slender, about 3.2× as long as broad, pedicel approximately 1.7× as long as broad and about 1.6× length of F1, F1 broader than long and approximately 1.3× length of F2, F3–F9 gradually increasing in length, F3–F5, F6–F7, and F8–F9 subequal in length, F10 longest flagellomere, approximately 1.5× as long as broad. Mesosoma approximately 1.2× as broad as long; mesoscutellum strongly convex, mediolongitudinal depression in apical half, giving bigibbous appearance (Fig. 6D). Forewing with two submarginal cells, first submarginal cell broader than second submarginal cell. Prolateral surfaces of metatibia and metabasitarsus each with a longitudinal carina (Fig. 6F). Metapretarsus with arolium (Fig. 6G). Metasoma faintly cordate (Fig. 6B) owing to concave anterior surface of T1 in dorsal view (Fig. 6A, B).

***Sculpturing and texture*.** Clypeus and paraocular area extending from base to antennal torulus with wrinkled punctures. Supraclypeal area and frons with coarse, dense punctures. Vertex with round punctures, punctures sparser than on supraclypeal area and frons. Mesoscutum with round, contiguous punctures, except polished and impunctate on short anterior-facing surface at medioapical margin; mesoscutellum with small, round, contiguous punctures (Fig. 6D). Metasomal T1–T3 with small, round, dense punctures; T4–T5 with small, round punctures, punctures sparser than those on T1–T3; T6 with wrinkled punctures.

***Color*.** Body black except as follows: clypeus yellow except black on apical margin; supraclypeal area with yellow laterally bordering subantennal sulci; paraocular area with yellow marking extending along inner margin to about lower tangent of antennal toruli. Frons with small yellow drop above medially (Fig. 6C). Gena with yellow mark above near vertex and posterior to upper margin of compound eye (Fig. 6E). Pronotal lobe yellow except small, round, dull spot medially, mesoscutum with thin, transverse, yellow markings latero-anteriorly on anterior border; axilla yellow (Fig. 6A). Prolateral surface of protibia yellow to yellowish brown. Metasomal T1 with yellow markings laterally, T3 basally yellow except broadly interrupted medially, T4–T5 with basal yellow bands, T6 yellow medially (Fig. 6B).

***Pubescence*.** Apical margin of clypeus with sparse, erect, yellow setae. Clypeus, supraclypeal area, and paraocular area with short, sparse, white to yellowish setae. Antennal scape with short, yellowish setae; above antennal toruli with long, dense, plumose tuft of diffuse white setae. Frons and vertex with short, sparse, yellowish setae. Anterior, posterior, and apical margin of mandible with short yellowish setae intermixed with some longer setae. Mesoscutum and mesoscutellum dorsally with short yellowish setae; mesoscutellum ventro-apically with longer, erect, yellow setae; propodeum with long, plumose, dense, yellowish setae. Prolateral surfaces of basitarsi and tarsi with dense, erect, yellowish setae; retrolateral surfaces of these same podites with tawny yellow setae. Metasomal T1 and T3–T5 with short, sparse, yellowish setae; T2 almost bare except some short yellowish setae laterally; T6 with short yellowish setae except dense, yellowish setae on apical margin; S2–S5 with long, dense, tawny yellow scopal setae.

♂: Latet.

##### Etymology.

The specific epithet is taken from the Latin adjective *flāvus* (meaning, “yellow”) and the noun *axilla* (meaning, “armpit” or “side”), and refers to the notable yellow markings on the axillae.

#### ﻿ Anthidiellum (Pycnanthidium) cornu

Taxon classificationAnimaliaHymenopteraMegachilidae

Tran, Engel & Nguyen sp. nov.

12348F20-F536-5459-8DF4-EE89CB2576C8

https://zoobank.org/CE0269DA-DE0A-483B-B8BA-CFA0E2FC3544

Figs 7A–G , 10C, D

##### Type material.

***Holotype*.** Vietnam: ♂, Kon Tum, Sa Thay, Sa Son, Chu Mom Ray NP, 14°25'19"N, 107°43'54"E, alt. 653 m, 25.iv.2022 [25 April 2022], Lien Thi Phuong Nguyen, Ngat Thi Tran leg. [IEBR]

***Paratype*.** Vietnam: 1♂, same data as holotype [IEBR].

##### Diagnosis.

The male of this species is similar to that of A. (P.) coronum. The new species differs from that species by the generally dull integument (integument shinier in *A.coronum*); the form of the male gonostylus in which the mesal surface of mesal branch bears some long setae and the mesal branch is long with its lateral margins slightly convex, while the outer branch is shorter, slender, and nearly straight, and the apical margins of both branches are straight (the mesal branch is long, swollen, with lateral margins curved, the outer branch is shorter, slender, and nearly straight, and the apical margin of both branches is rounded in *A.coronum*).

##### Description.

♂: body length 7–7.2 mm (holotype = 7.0 mm), forewing length 6.3–6.5 mm (holotype = 6.5 mm).

**Figure 7.**
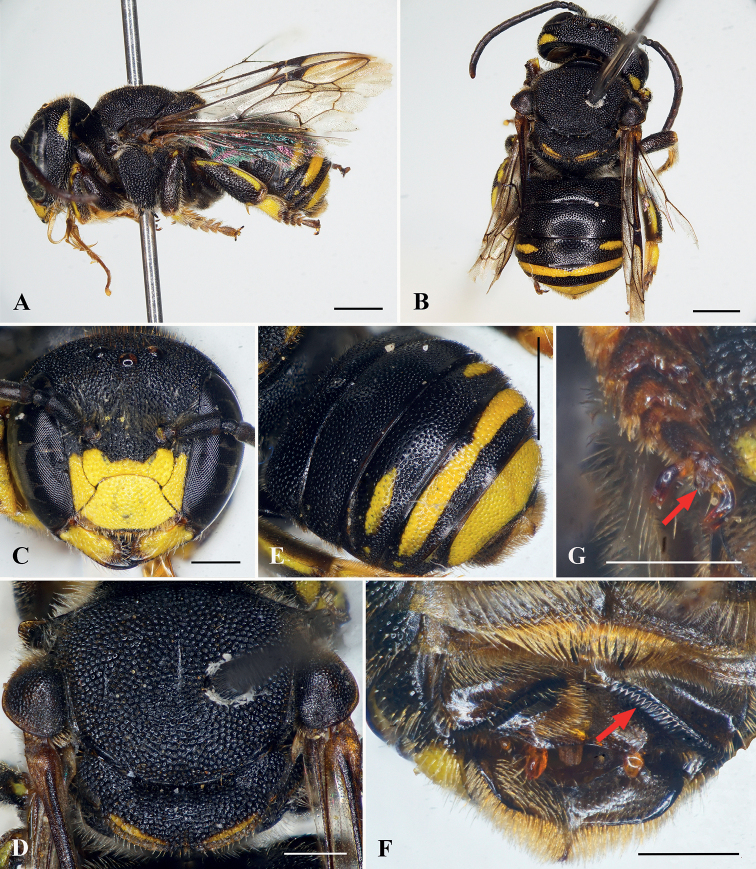
Anthidiellum (Pycnanthidium) cornu Tran, Engel & Nguyen, sp. nov., holotype, male **A** lateral habitus **B** dorsal habitus **C** facial view **D** mesosoma in dorsal view **E** oblique dorsal view of metasoma **F** metasomal apex, red arrow indicating stiff peg-like and scale-like setae on apical margin of metasomal S5 **G** metapretarsus, red arrow indicating arolium. Scale bars: 1 mm (**A, B, F**); 0.5 mm (**C–E, G**).

***Structure*.** Head broader than long, about 1.2× as broad as long (Fig. 7C) Compound eyes approximately 2.1× as long as broad, about 1.7× genal width. Mandible tridentate, first two teeth short and equal in length, third tooth longest (Fig. 7C). Clypeus flat, 1.8× as broad as long. Supraclypeal area slightly convex. Antennal scape short, about 2.8× as long as broad, pedicel approximately as broad as long and about 1.3× length of F1, F1 broader than long and approximately 0.4× length of F2, F2–F9, subequal in length, F10 longest flagellomere, 2.5× as long as broad. Mesoscutum about 1.2× as broad as long; mesoscutellum slightly convex, with mediolongitudinal depression apically (Fig. 7D). Forewing with two submarginal cells, first submarginal cell broader than second submarginal cell. Metatibia and metabasitarsus enlarged (Fig. 7A); metapretarsal arolium present (Fig. 7G). Anterior surface of metasomal T1 concave; T7 with a short spine medially (Fig. 7E); apical margin of S5 with comb of stiff, black peg-like and scale-like setae (Fig. 7F). Male genitalia with gonostylus forked into two unequal branches, mesal surface of mesal branch with some long setae, mesal branch long, lateral margins slightly convex, outer branch shorter, slender, nearly straight, apices of both branches straight, transverse (Fig. 10C, D).

***Sculpturing and texture*.** Mandible with shallow, sparse punctures. Clypeus, basal half of supraclypeal area, and paraocular area from base to lower tangent of antennal toruli with shallow, dense, wrinkled punctures; remainder of supraclypeal area with coarse, dense punctures. Frons with coarse, contiguous punctures. Vertex with round, dense punctures. Mesoscutum with coarse, contiguous punctures, punctures larger than those on frons; mesoscutellum with coarse, contiguous, wrinkled punctures, punctures smaller than those on mesoscutum. Metasomal T1–T2 with small, round, dense punctures; T3–T5 with sparse punctures basally, blending to dense, wrinkled punctures on remainder of terga; T6 with shallow, dense punctures.

***Color*.** Body dull black except as follows: mandible yellow except brownish black apically; clypeus yellow except tawny yellow on apical margin; paraocular area with yellow extending along inner ocular margin to lower tangent of antennal toruli; supraclypeal area yellow apically and laterally; antennal space apico-ventrally with small, dull yellow-orange marking; gena with yellow markings posterior to upper border of compound eye, extending from near tangent with top of eye to exceeding tangent (Fig. 7A, C). Mesoscutellum with thin, dull yellow marking on apical margin and interrupted medially (Fig. 7B). Profemur apically with small yellow mark; prolateral surface of protibia with thin, yellow mark; mesotibia with small yellow mark basally; superior margin of prolateral surface of metatibia with thin, yellow mark; entire metabasitarsus yellow. Metasomal T3 with yellow band interrupted medially; T4 with yellow band (interrupted medially in paratype), T5–T6 with yellow, T7 with yellowish mark medially (T7 blackish in paratype) (Fig. 7B, E).

***Pubescence*.** Outer surface and dorsal and apical margins of mandible with short, sparse, yellowish setae; ventral margin of mandible with short, yellowish setae intermixed with longer setae. Apical margin of clypeus with some short, yellowish setae; clypeus laterally, paraocular area, and basal half of supraclypeal area with some sparse, short, yellowish setae. Antennal scape with short yellowish setae; face above antennal torulus with tuft of long, yellowish, plumose setae. Vertex with sparse, short, yellowish setae. Mesoscutum and mesoscutellum with short, yellowish setae. Propodeum with dense, long, plumose, white setae. Prolateral surfaces of metabasitarsus and metatarsus with dense, erect, white setae; retrolateral surfaces of these same podites with tawny yellow setae. Metasomal S1 with short, white tuft of setae on apical margin; S2 with longer, white tuft of setae on apical margin; S3 with long, tawny yellow tuft of setae on medioapical margin; S4 with yellowish tuft of setae on apical margin; surface of S6 with long, yellowish setae, apical margin of S6 with long, yellowish, plumose setae.

##### Etymology.

The specific epithet is the Latin noun *cornū* (meaning, “horn” or “antler”) and refers to the shape of the gonostyli of the male genitalia, which superficially resemble antlers.

#### ﻿ Anthidiellum (Pycnanthidium) coronum

Taxon classificationAnimaliaHymenopteraMegachilidae

(Wu, 2004)

524AE018-EC90-5EAA-95C9-3D6A09F15EB7

**Figure 8.**
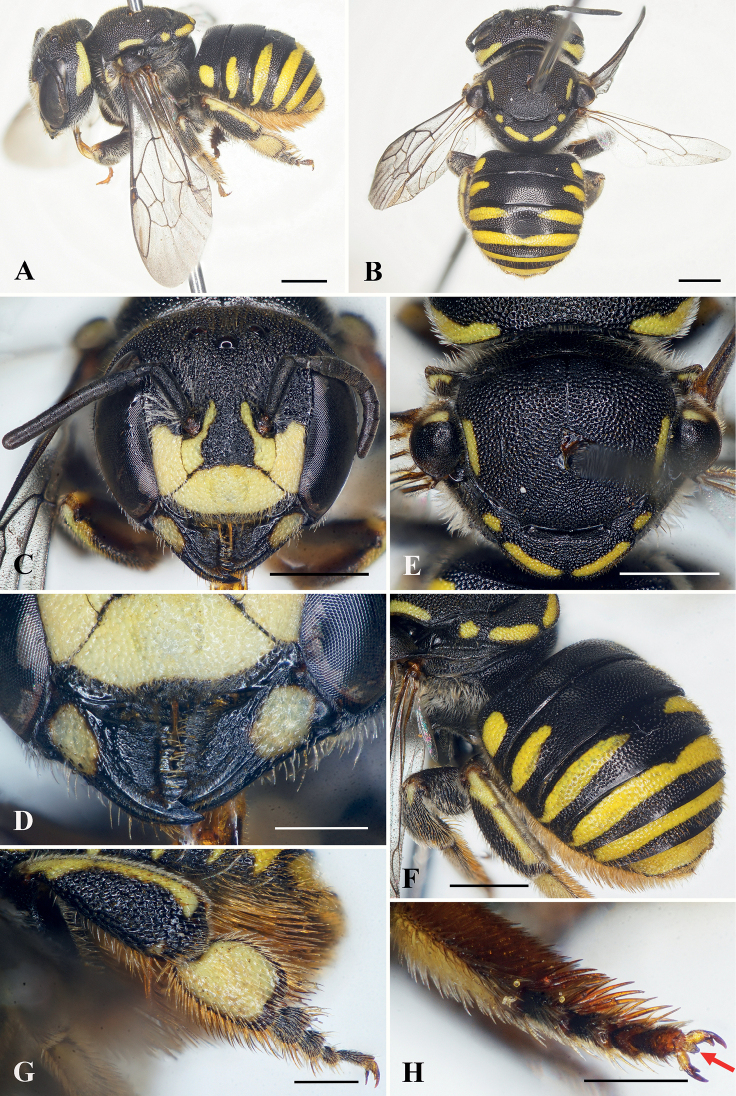
Anthidiellum (Pycnanthidium) coronum (Wu, 2004), female **A** lateral habitus **B** dorsal habitus **C** facial view **D** mandibles **E** mesosoma in dorsal view **F** dorsal oblique view of metasoma **G** prolateral surfaces of metatibia and metatarsus **H** metatarsus and metapretarsus, red arrow indicating arolium. Scale bars: 1 mm (**A–C, E, F**); 0.5 mm (**D, G, H**).

**Figure 9.**
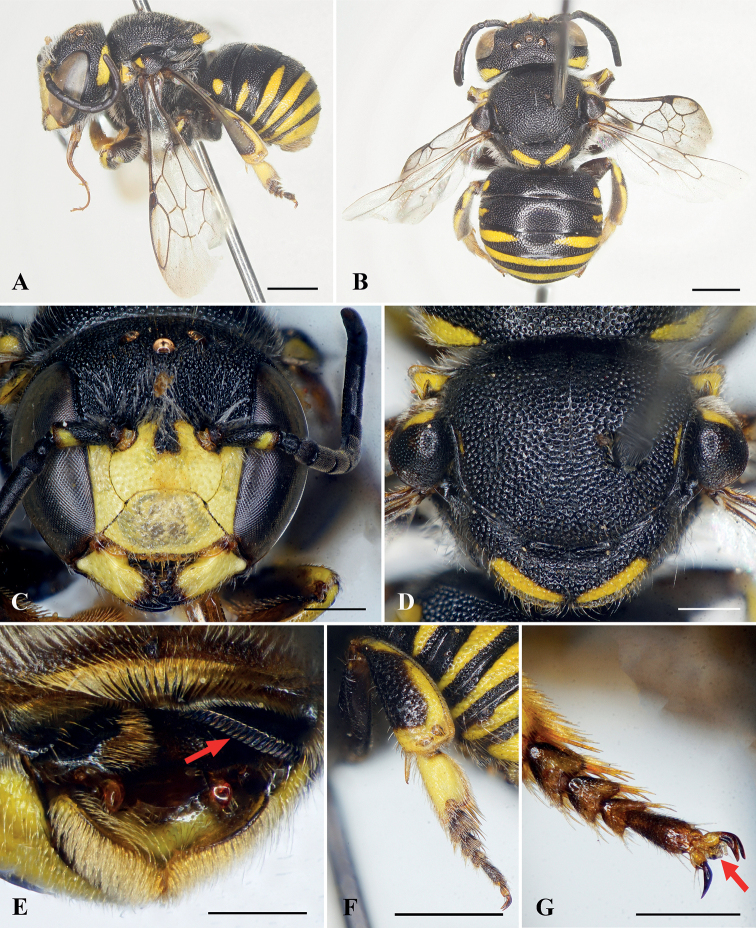
Anthidiellum (Pycnanthidium) coronum (Wu, 2004), male **A** lateral habitus **B** dorsal habitus **C** facial view **D** mesosoma in dorsal view **E** metasomal apex in ventral view, red arrow indicating thick peg-like and scale-like setae **F** prolateral surfaces of metatibia and metatarsus **G** metadistitarsus and metapretarsus, red arrow indicating arolium. Scale bars: 1 mm (**A, B, D, F**); 0.5 mm (**C, E, G**).

**Figure 10.**
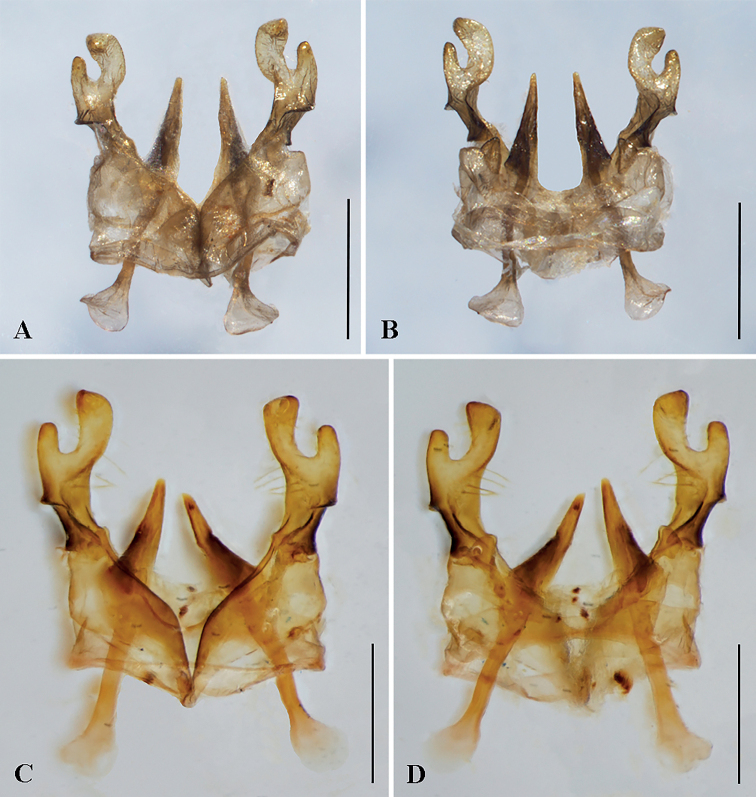
Male genitalia of two Vietnamese species of *Anthidiellum* Cockerell (Anthidiini) (dorsal views at left, ventral views at right) **A, B**Anthidiellum (Pycnanthidium) coronum (Wu, 2004) **C, D**A. (P.) cornu Tran, Engel & Nguyen, sp. nov. Scale bars: 0.5 mm.

Figs 8A–H , 9A–G , 10A, B

Trachusa (Paraanthidium) coronum Wu, 2004: 545 [holotype ♀]; Wu 2006: 177.
Anthidiellum (Pycnanthidium) coronum (Wu); Niu et al. 2016: 337.

##### Material examined.

Vietnam: 1♂, Gia Lai, Mang Yang, Ayun, Kon Ka Kinh NP, 14°12'11"N, 108°18'58"E, alt. 834 m, 25.iii.2022 [25 March 2022], Lien Thi Phuong Nguyen, Ngat Thi Tran, Cuong Quang Nguyen leg. [IEBR]; 3♀♀2♂♂, Gia Lai, Mang Yang, Ayun, Kon Ka Kinh NP, 14°12'11"N, 108°18'58"E, alt. 834 m, 26.iv.2022 [26 April 2022], Lien Thi Phuong Nguyen, Ngat Thi Tran leg. [2♀♀2♂ in IEBR, 1♀ in AMNH]; 1♂, Gia Lai, Mang Yang, Ayun, Kon Ka Kinh NP, 14°12'11"N, 108°18'58"E, alt. 834 m, 27.iv.2022 [27 April 2022], Lien Thi Phuong Nguyen, Ngat Thi Tran leg. [AMNH]

##### Description

**(hitherto undescribed).** ♂: body length 7.0 mm, forewing length 6.0 mm.

***Structure*.** Head broader than long, about 1.2× as broad as long (Fig. 9C). Compound eyes about 2.1× as long as broad, about 1.7× genal width. Mandible tridentate, first two teeth short, subequal in length, third tooth longest (Fig. 9C). Clypeus flat, about 1.8× as broad as long. Supraclypeal area slightly convex. Antennal scape short, about 3× as long as broad, pedicel about 1.3× as broad as long and subequal in length of F1, F1 short, broader than long and approximately 0.6× length of F2, F2–F9 subequal in length, F10 longest flagellomere, about 2.2× as long as broad. Mesoscutum approximately 1.2× as broad as long; mesoscutellum slightly convex, with mediolongitudinal depression apically (Fig. 9B). Forewing with two submarginal cells, first submarginal cell broader than second submarginal cell. Metatibia and metabasitarsi enlarged (Fig. 9F); metapretarsal arolium present (Fig. 9G). Anterior surface of metasomal T1 concave; T7 with a short spine medially; apical margin of S5 with comb of stiff, black, peg-like setae (Fig. 9E). Male genitalia with gonostylus forked into two unequal branches, inner branch long, swollen, lateral margins curved, outer branch shorter, slender, nearly straight (Fig. 10A, B).

***Sculpturing and texture*.** Mandible from base with sparse, faint, wrinkled punctures except small punctures apically. Clypeus, supraclypeal area, and paraocular area from base to above tangent above antennal toruli with shallow, wrinkled punctures. Frons with coarse, dense punctures. Vertex with round, dense punctures, punctures larger than those on frons. Mesoscutum with round, dense punctures, punctures larger than those on vertex; mesoscutellum with dense, wrinkled punctures (Fig. 9D). Metasomal T1–T2 with small, round punctures; T3–T5 with sparse punctures basally and dense punctures on remainder of terga; T6 with shallow, dense punctures.

***Color*.** Body black except as follows: clypeus yellow except yellow brown transparent on apical margin; mandible yellow except brownish black on apically; paraocular area with yellow marking extending along the inner margin to above the antennal socket; supraclypeal area with yellow marking as Fig. 8C; antennal space apico-ventrally with small yellow marking; gena with yellow markings extending from the two-third of the eye to exceed the top of eye (Fig. 9C). Pronotal lobe yellow, except yellow transparent in apical margin and a yellow brown transparent spot on the median area; in dorsal view, mesoscutum lateral margin with thin, short, paint yellow markings (or absent in some specimens); tegula basally with yellow markings; mesoscutellum with yellow marking and interrupted on apico-medially, apical margin of yellow to yellow to yellow transparent marking; axilla black (or with small, yellow spot in some specimens) (Fig. 9D). Profemur latero-apical with small yellow markings; outer surface of protibia and mesotibia with thin, yellow marking, posterior and apical margins of metatibia with yellow marking and presence of two small brown marks embedded on yellow marking apically; all basitarsus yellow, tarsi dull tawny yellow. Metasomal T1–T2 laterally with small yellow markings, T3 basally with yellow band and interrupted medially, T4–T5 basally with yellow bands, T6–T7 with yellow, except baso-laterally (Fig. 9A, B).

***Pubescence*.** Clypeus with some short, yellowish setae on apical margin; paraocular area and supraclypeal area with some sparse, short, yellowish setae; scape with short yellowish setae; face above antennal torulus and frons with tufts of long, white, plumose setae (Fig. 9C); vertex with sparse, short, yellowish setae. Margins of mandible with short, yellowish setae intermixed with some longer setae. Mesoscutum and mesoscutellum with short yellowish setae. Propodeum with dense, long, plumose, white setae. Prolateral surfaces of metabasitarsus and metatarsus with dense, erect, white setae; retrolateral surfaces of these same podites with tawny yellow setae. Metasomal S3 medio-apical margin with tuft of long, tawny yellow setae; surface of S6 with long, yellowish setae and apical margin of S6 with long, yellowish, plumose setae.

### ﻿Key to species of *Anthidiellum* from Vietnam

Characters for the key were extracted from the original descriptions of Wu (1962, 2004); the females of A. (P.) cornu and males of A. (C.) nahang, A. (P.) ayun, A. (P.) chumomray, and A. (P.) flavaxilla, remain unknown.

**Table:** 

1	Male	**2**
–	Female	**4**
2	Metatibia and metabasitasus not enlarged, prolateral surface of each with a longitudinal carina; mesoscutum with transverse yellow marks anterolaterally on anterior border	**A. (P.) carinatum (Wu, 1962)**
–	Metatibia and metabasitasus enlarged, prolateral surface of each without a longitudinal carina; mesoscutum without yellow marks anterolaterally	**3**
3	Metasomal T1–T2 with yellow laterally; gonostylus of male forked into two unequal branches, apical margin of both branches rounded, inner branch strongly curved outward laterally; integument shiny	**A. (P.) coronum (Wu, 2004)**
–	Metasomal T1–T2 black; gonostylus of male forked into two unequal branches, apical margin of both two branches straight, inner branch slightly convex laterally; integument generally dull	**A. (P.) cornu Tran, Engel & Nguyen, sp. nov.**
4	Tegula narrowly rounded posteriorly; omaular carina extending along only upper half of omaular angle; mesoscutellum less sharply margined, with distinct mediolongitudinal depression apically; forewing bicolorous, rather clear proximally and infumate apically; metasomal T5–T6 yellow	**A. (C.) nahang Tran, Engel & Nguyen, sp. nov.**
–	Tegula broadly rounded or almost transverse posteriorly; omaular carina extending to venter (in some *Pycnanthidium* weak or even absent on lower half of mesepisterum); mesoscutellum sharply margined, with narrow mediolongitudinal depression apically; forewing not bicolorous; nearly all metasomal terga with yellow markings	**5**
5	Metatibia and metabasitasus not enlarged, prolateral surface of each with a longitudinal carina; metasomal T2 black	**6**
–	Metatibia and metabasitasus enlarged, prolateral surface of each without a longitudinal carina; metasomal T2 yellow laterally	**A. (P.) coronum (Wu, 2004)**
6	Supraclypeal area, pronotal lobe, and axilla all marked with yellow	**7**
–	Supraclypeal area, pronotal lobe, and axilla black	**8**
7	Medioapical margin of mesoscutum nearly straight, densely punctate up to margin; mesoscutellum with yellow on apicolateral margins	**A. (P.) carinatum (Wu, 1962)**
–	Medioapical margin of mesoscutum vertical, vertical surface polished and impunctate medially; mesoscutellum black	**A. (P.) flavaxilla Tran, Engel & Nguyen, sp. nov.**
8	Clypeus approximately 1.4× as broad as long; paraocular area black; mesoscutellum convex and bigibbous apically	**A. (P.) ayun Tran, Engel & Nguyen, sp. nov.**
–	Clypeus 1.6× as broad as long; paraocular area yellow to lower tangent of antennal toruli; mesoscutellum flat	**A. (P.) chumomray Tran, Engel & Nguyen, sp. nov.**

## ﻿Discussion

Previously, two species of *Anthidiellum* were recorded from the northern provinces of Vietnam (Khuat et al. 2012). Khuat et al. (2012) identified their material as *Anthidiellumrasorium* (Smith, 1875) (Phu Tho Province, label “Apoi.0237”) and *Anthidiellum* sp. (Bac Giang Province) (deposited in the collection of Hymenoptera of IEBR). We examined the material reported by Khuat et al. (2012) and the material of the former species is a specimen of *Heriades* while the material of the latter is now missing. Given these misidentifications, the genus *Anthidiellum* had not been formally documented from Vietnam until the present report, even though the genus must have occurred here based on all of the extralimital records that implied a distribution for the clade across northern Vietnam, at the least. It is nice to be able to formally demonstrate the occurrence of the genus within the country, based on a small diversity of species.

**Figure 11.**
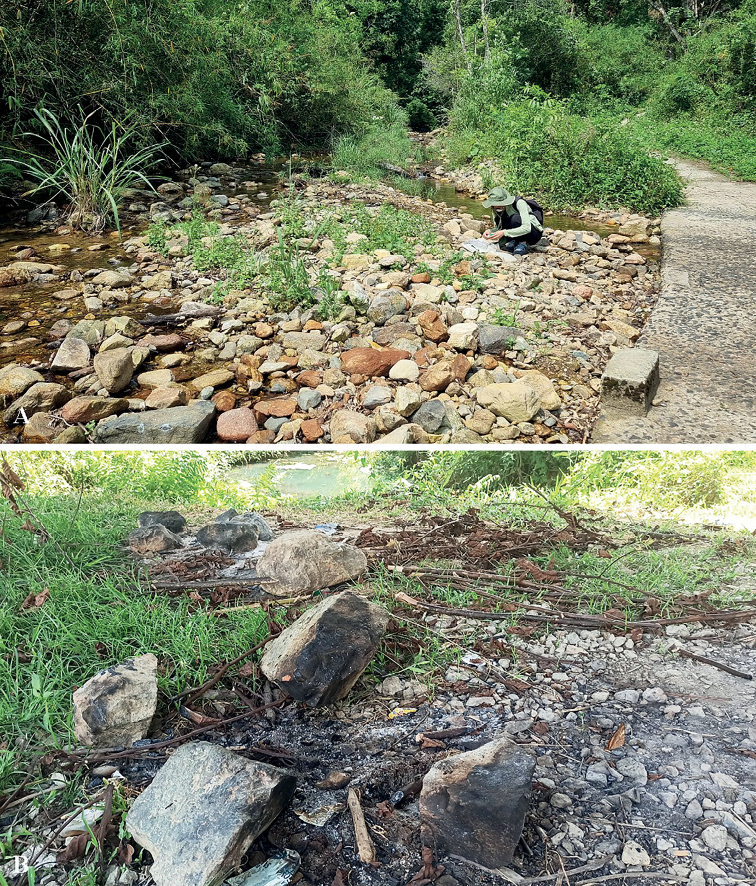
Habitats of Vietnamese species of *Pycnanthidium* Krombein **A** rocky area along a stream **B** pile of ash.

Considering the habitats in which the species were encountered, all Vietnamese species of *Pycnanthidium* were found on small rocks or collected in ash piles from the burning of small logs. Furthermore, all of the localities were next to small streams (Fig. 11). It seems likely that the bees were looking for a source of mineral salts and water at the time they were captured. It is greatly hoped that future studies can explore the biology of these species, ideally locating nests and discovering immature stages, nest associates, and floral associations.

## Supplementary Material

XML Treatment for Anthidiellum (Clypanthidium) nahang

XML Treatment for Anthidiellum (Pycnanthidium) ayun

XML Treatment for Anthidiellum (Pycnanthidium) carinatum

XML Treatment for Anthidiellum (Pycnanthidium) chumomray

XML Treatment for Anthidiellum (Pycnanthidium) flavaxilla

XML Treatment for Anthidiellum (Pycnanthidium) cornu

XML Treatment for Anthidiellum (Pycnanthidium) coronum
